# Associations among Physical Activity, Diet, and Obesity Measures Change during Adolescence

**DOI:** 10.1155/2015/805065

**Published:** 2015-10-11

**Authors:** Janne H. Maier, Ronald Barry

**Affiliations:** ^1^University of Alaska Fairbanks, P.O. Box 757500, Fairbanks, AK 99775, USA; ^2^Institute of Arctic Biology, University of Alaska Fairbanks, P.O. Box 757500, Fairbanks, AK 99775, USA; ^3^Center for Alaska Native Health Research, University of Alaska Fairbanks, P.O. Box 757500, Fairbanks, AK 99775, USA; ^4^Department of Mathematical Sciences, University of Alaska Fairbanks, P.O. Box 757500, Fairbanks, AK 99775, USA

## Abstract

*Background*. Obesity in youth is highly prevalent. Physical activity and diet are influential in obesity development. However, there is a knowledge gap regarding links between activity and diet quality and their combined influence on obesity during adolescence. *Objectives*. We used five years of data from 2379 adolescent girls in the National Heart Lung and Blood Institute Growth and Health Study to evaluate the association between physical activity and diet quality during adolescence and to assess both as correlates of obesity. *Design*. Diet, activity, and body composition measures were evaluated pairwise for correlation. A canonical correlation analysis was used to evaluate relationships within and between variable groups. All statistics were examined for trends over time. *Results*. We found positive correlations between physical activity and diet quality that became stronger with age. Additionally we discovered an age-related decrease in association between obesity correlates and body composition. *Conclusion*. These results suggest that while health behaviors, like diet and activity, become more closely linked during growth, obesity becomes less influenced by health behaviors and other factors. This should motivate focus on juvenile obesity prevention capitalizing on the pliable framework for establishing healthy diet and physical activity patterns while impact on body composition is greatest.

## 1. Introduction

The 2009-2010 National Health and Nutrition Examination Survey (NHANES) found United States juvenile obesity (Body Mass Index (BMI) ≥ 95th percentile in BMI-for-age growth charts) at 16.9% and adult male/female obesity (BMI ≥ 30) at 35.5%/35.8%, respectively [1, 2]. While the high adult obesity prevalence is daunting, more concerning is the commonness of obesity in youth because juvenile obesity tracks into adulthood and is a precursor for obesity related diseases [3].

Current USDA physical activity guidelines for weight management advise a minimum of 60 minutes/day for ages 6–17 and 150 minutes/week of moderate activity or 75 minutes/week of moderate/vigorous activity for adults [4]. Accelerometer measures of physical activity, including occupational and transportation activity, from 2003-2004 NHANES data show that adherence to physical activity recommendation is 42% for 6–11-year-olds, 8% for 12–19-year-olds, and less than 5% for adults [5]. The suboptimal adherence to recommended physical activity, and the drastic decrease in physical activity during adolescence [5], particularly in girls [6], is directly correlated with the current rise in obesity [7–9].

Diet recommendations for weight management are limiting calorie intake and increasing diet quality by choosing fruits, vegetables, and foods high in fiber and low in sugar and saturated fat [4, 10]. In the industrialized world, diet quality may be assessed by using dietary energy density (kilocalories/gram) as a proxy because micronutrient intake, especially from fruits and vegetables, and diet quality are negatively correlated with dietary energy density [11, 12]. Most adults and children exceed suggested total and saturated fat intake [13, 14] in spite of a decline in intake of energy, fat, and saturated fat in the last decades of the 20th century [13], and adolescent girls are singled out as the only group in NHANES for whom energy intake has increased over this period [13].

While associations between physical activity and diet are well reported in adults and youths [15, 16], less is known about how they interact during adolescence. Diet and activity patterns change during adolescence [17], so it would be pertinent to examine potential changes in associations between these behaviors and their relationship to body composition during this time.

We report results from a secondary analysis on diet and physical activity associations using five years of data spanning seven years of development in adolescent girls from The National Heart Lung and Blood Institute Growth and Health Study (NGHS). We also investigated both diet and physical activity as correlates of body composition (BMI and body fat percent) during growth. Understanding the interactions and underpinnings of diet and physical activity is paramount to stemming the obesity epidemic, and identifying changes in associations during development may pinpoint an optimal intervention window for preventing adult obesity.

## 2. Subjects and Methods

### 2.1. The Growth and Health Study

The National Heart Lung and Blood Institute Growth and Health Study was conducted to evaluate racial differences in the development of obesity and CVD risk in girls. The study group recruited 1213 black girls and 1166 white girls from schools and clinics in the study areas, Berkeley, CA, Cincinnati, OH, and Rockville, MD, from January 1987 to May 1988 [18]. To be eligible for the study, the girls had to be white or black with no other mixed heritage. The girls were all 9-10 years old at recruitment (visit 1) and attended annual visits for 10 years with a follow-up rate of 89% [19]. The centers collected annual anthropometric measures (e.g., height, weight, skin fold thicknesses, and maturation stage indicators), as well as dietary and physical activity information. Extensive sampling design and study methods are described elsewhere [18, 20]. Our analysis to assess associations between diet and exercise and to identify correlates of obesity during adolescence in this cohort was approved by University of Alaska Institutional Review Board (ID# 231373-4). Subsequently, the National Heart Lung and Blood Institute's Biologic Specimen and Data Repository Information Coordinating Center (BioLINCC) approved the analysis objectives and fulfilled the NGHS data request. This paper was prepared using research materials obtained through BioLINCC, but it does not necessarily reflect the opinions or views of the NGHS or the National Heart Lung and Blood Institute.

### 2.2. Physical Activity and Diet Measures

In the NGHS, information about physical activity levels was collected using two questionnaires validated for use in children, a habitual activity questionnaire (HAQ) and an activity diary (AD) [18, 20]. The HAQ asked participants to report type and frequency of activities in school and outside of school throughout the year. The HAQ was interviewer administered in visit years 1, 3, and 5, and it was self-administered years 7–10. The weekly scores were calculated by multiplying the weekly frequency of the activity by the fraction of year that the activity was engaged in and by the metabolic equivalent of task (MET) value for the activity. Weekly MET scores for reported activities for the previous year were added to yield the annual HAQ score as an estimate of physical activity throughout the year [20]. In visit years 1–5, 7, 8, and 10, the participant completed an AD on three consecutive days along with a 3-day food journal. The participants were instructed to record both active (e.g., jogging, kickball, and jumping rope) and sedentary (e.g., sitting to talk, watching TV, or reading) behaviors listed in their AD in designated timeslots. Along with the listed activity categories there was a blank “other activities” section to document activities not listed in the questionnaire. Daily AD scores were calculated by multiplying the MET value for each activity by its duration and summing the calculated MET scores for all the activities in one day. Final AD score was the average of the scores for all the usable days. The participant reviewed the AD and HAQ with staff at the centers using a common protocol for submission in each collection year [20].

Information about nutrient and calorie intake was collected with the 3-day food journal each year [18, 20]. The journals were completed by the participants on two consecutive weekdays and one weekend day, and the journal entries were reviewed and confirmed by the participant with staff following standardized protocol for all collection centers. Food journals were coded centrally and processed to yield information about average kilocalorie (kcal) intake, macronutrient distribution, and average intake of 50 different nutrients. During years 1 and 2 the data were processed at the Nutrition Coordinating Center in Minnesota, and the records from years 3 through 10 were processed at the NGHS Dietary Data Entry Center in Cincinnati [21].

Anthropometrics like height, weight, and skin fold measures were collected at annual visits [18]. The average of two measures was taken, and if the two measures deviated from each other excessively (more than 0.5 cm for height, 0.3 kg for weight, and 1.0 mm for skin folds), a third measure was included [18]. Maturation was assessed with a modified Tanner staging method to accurately gauge maturation at different body compositions [18].

### 2.3. Analysis

We used data from years 3, 5, 7, 8, and 10 because these years had physical activity information collected by both HAQ and AD ensuring a comprehensive physical activity assessment. We started at year three because data from this and subsequent years were processed at the Cincinnati Dietary Data Entry Center. These data include both total kcal and grams of intake which are needed for energy density calculations. Daily energy density for each journal period was calculated by dividing the total daily kcal by the daily sum of gram intake of all reported foods and drinks; energy density was then averaged for all valid journal days. To indicate diet quality we used average energy density, average kcal intake, average dietary saturated fat percent, and average (gram) fiber intake. To assess body composition we included BMI calculated from height and weight measurements and body fat percent estimated from skin fold measurements; in the NGHS body fat percent was calculated with a standard equation using measurements of triceps, subscapular, and suprailiac skin folds [18]. Household income, race, maturation stage, height, and weight were included to adjust for confounding effects. To account for racial differences in the recording of physical activity measures, we included variables for racial interaction with HAQ (race × HAQ) and racial interaction with AD (race × AD) in the analyses. All statistical analyses were conducted with SPSS 22 [22].

Initially we ran bivariate correlation analyses between all variables pairwise. To account for simultaneous evaluation of multiple variables we only considered *P* < 0.001 as significant. The NGHS dataset is large, so we used Spearman's rho correlation because it is less sensitive to potential outliers than Pearson's *r*.

To examine relationships between variable groups, we conducted a canonical correlation analysis (CCA). Canonical correlation is a multivariate approach where a program constructs linear combinations of two sets of variables:

 Canonical variates (*X*):
  (1) $$\begin{array}{c} a_{1}X_{i1}+a_{2}X_{i2}+\cdots +a_{p}X_{ip}=U_{i}. \end{array}$$
 Canonical covariates (*Y*):
  (2) $$\begin{array}{c} b_{1}Y_{i1}+b_{2}Y_{i2}+\cdots +b_{q}Y_{iq}=V_{i}. \end{array}$$

The linear indices are adjusted so that, for the *i*th set of observations, the correlation between the two resulting latent values, *U* and *V*, is maximized. The latent values represent underlying structure in the data. Statistics from CCA include several index sets, the first set of which explain the largest proportion of variance in the data and have the highest canonical correlation between variates and covariates. The canonical variates are evaluated for significance in the analysis with univariate *F* tests. Data from CCA can be interpreted by the canonical correlation (values ranging from 0 to 1), a measure of correlation between the latent values from the two linear indices. Additionally, the individual coefficients in each index tell us how the variables are related to each other within the group when the groups are most correlated with each other.

To evaluate the association between physical activity and diet quality we grouped variables for CCA as follows. Canonical variates (physical activity and confounders) were household income, race, height, weight, maturation stage, smoking status, race × HAQ, race × AD, HAQ, and AD, and canonical covariates (diet quality indicators) were average energy density, average kcal, average dietary saturated fat percent, and average fiber intake.

Using CCA to evaluate correlates of obesity during growth was done by grouping the variables by household income, race, maturation stage, smoking status, race × HAQ, race × AD, HAQ, AD, average energy density, average kcal, average dietary saturated fat percent, and average fiber intake (obesity correlates) as canonical variates and BMI and body fat percent (body composition) as canonical covariates; height and weight were not included as variates in the second part of the analysis as they are direct predictors of BMI and including them gave an artificially high correlation. To allow evaluation of significance with regard to canonical correlation, the confounding variables were grouped as variates in this analysis. Statistics from all visit years were compared to identify trends during adolescence.

## 3. Results

### 3.1. Associations between Physical Activity and Diet

**Figure 1:**
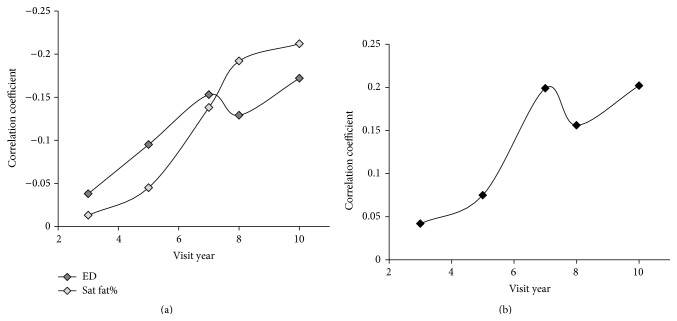
Spearman's rho correlation coefficients. (a) Correlation between habitual physical activity and average energy density (ED) and dietary saturated fat percent (Sat fat %) by visit year. (b) Correlation between habitual physical activity and fiber intake by visit year. Correlation is significant (two-tailed *t*, *P* < 0.001) for years 5, 7, 8, and 10 for correlation with ED and fiber, and for years 7, 8, and 10 for correlation with Sat fat %.

Figure 1(a) shows that the strength of the negative correlation, as indicated by Spearman's rho, between HAQ scores and dietary energy density and between HAQ scores and dietary saturated fat percent increased with age. The increase followed a linear trend with *R* ^2^ = 0.89 for energy density and *R* ^2^ = 0.95 saturated fat percent.  Figure 1(b) shows that the strength of the positive correlation, as indicated by Spearman's rho, between HAQ scores and fiber intake also increased with age (*R* ^2^ = 0.82).

Examining association between physical activity and diet with CCA initially produced opposite coefficients for the two physical activity measures. Spearman's rho correlations between HAQ and 3d AD scores indicated that the measures were positively correlated with each other at each visit year (*P* < 0.001), but scores for HAQ had a positive coefficient and scores for 3d AD a negative coefficient for all years in the initial CCA results. Data stratification by race revealed racial differences in responses on physical activity questionnaire, so racial interaction variables were added as confounders in the CCA.

Table 1 presents standardized canonical correlation coefficients from the first index in the CCA examining association between physical activity and diet including the racial interaction variables with the confounders. The first index explained 66% to 75% of the variance in the data. Maturation stage, height, and weight were included as confounding variables in the analysis but their coefficients are not reported in Table 1. In visit years 3 and 5 HAQ scores and saturated fat percent had negative and positive coefficients, respectively, and positive and negative coefficients, respectively, for years 7–10, but for the rest of the variables, the coefficients are consistent from year to year. Correlation coefficients for visits 7, 8, and 10 indicate that higher household income, being white (white = 1, black = 2), and high physical activity scores yield the highest canonical correlation to low dietary energy density, low kcal intake, and low dietary saturated fat percent and high intake of fiber.

**Figure 2:**
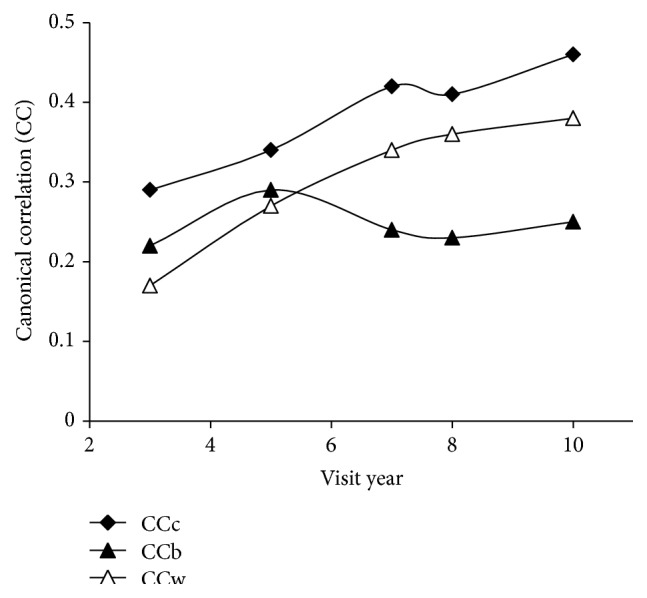
Canonical correlation of physical activity and confounders with diet quality indicators by visit year. Physical activity and confounders: income category, race, maturation stage, height, weight, habitual physical activity questionnaire score (HAQ), 3-day activity diary score (3d AD), racial interaction HAQ, and racial interaction 3d AD; diet quality indicators: average energy density, average caloric intake, average dietary saturated fat percent, and average fiber intake. Lines represent stratified data from black (CCb) and white (CCw) girls and combined (CCc) data.

As illustrated in Figure 2, the canonical correlation of physical activity scores and confounding variables with diet quality indicators increases with time for the combined data and for white girls only. The correlation increase in Figure 2 follows a linear trend with *R* ^2^ = 0.95 for the combined data and *R* ^2^ = 0.92 for white girls. The canonical correlation for black girls does not mimic the trend for the combined data or for white girls. The correlation of physical activity scores and confounders with diet quality in black girls increases from years 3 to 5 but shows a sharp decrease in year 7, the year the HAQ went from interview to self-administered, which may indicate reporting bias. From years 8 to 10 there is a weak increase in correlation for association of physical activity and confounders with diet quality in black girls indicating adherence to the general trend after adjusting to the change in recording methods.

### 3.2. Correlates of Obesity during Growth

Bivariate analysis examining correlates of obesity shows that household income, HAQ scores, and fiber intake are negatively correlated with BMI and body fat percent (Spearman's rho correlation coefficients vary from −0.06 to −0.17), and race was positively correlated with both obesity measures (Spearman's rho correlation coefficients vary from 0.09 to 0.21) in every year (*P* < 0.001) indicating that being black was correlated with higher BMI and body fat percent. The strength of the correlation for BMI and body fat percent with household income, race, and HAQ increased a little during the study years (increase range: 0.019 to 0.129), but the correlation of BMI and body fat percent with AD and diet quality variables did not increase and the sign, strength, and significance of the coefficients varied from year to year.

We conducted CCA between body composition (BMI and body fat percent) and obesity correlates (physical activity, diet quality indicators, and confounders) to identify important obesity correlates and to consider trends in correlation between the two during adolescence.  Table 2 provides the standardized canonical correlation coefficients from the first index for canonical correlation between obesity correlates and body composition from this CCA. BMI is a measure of body fatness, but there are inverse coefficients for BMI and body fat percent. This is an effect of the two correlated variables simultaneously explaining variance in body composition. Correlation coefficients for obesity correlates indicate that higher income and lower race score (white) consistently yielded the highest canonical correlation with body composition; however, the relationships between the other obesity correlates varied by year. For example, from years 5 to 7, when the groups are most correlated, the coefficients for activity measures, kcal, saturated fat percent, and fiber all changed signs (positive and negative). Nevertheless, some variables remained significant regardless of change in signs of the coefficients. In addition to income, race, and racial interaction variables, both activity measures and fiber intake were significant for canonical correlation with obesity measures for most (3d AD) or all (HAQ, fiber) visit years in the analysis. Other diet quality variables except energy density were significant for at least three of the five study years. In this CCA, the first index explained 62% to 84% of the variance in the data (Table 2) indicating that income, race, activity, and diet are all important obesity correlates.

**Figure 3:**
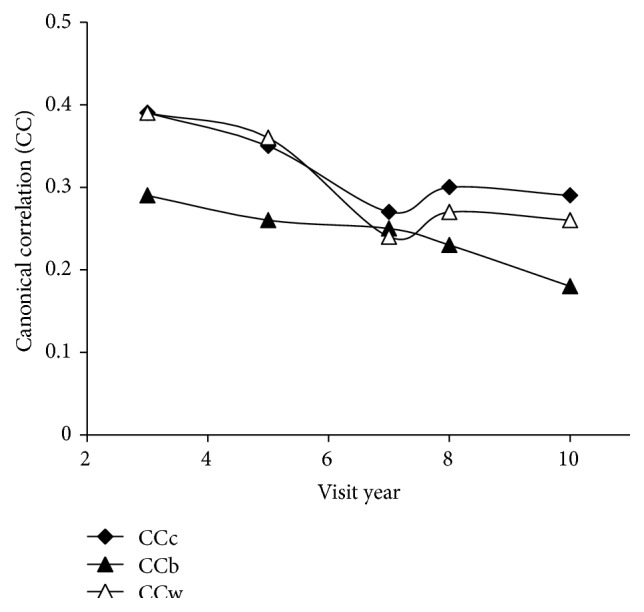
Canonical correlation between obesity correlates and body composition by visit year. Obesity correlates: income category, race, maturation stage, habitual physical activity questionnaire score (HAQ), 3-day activity diary score (3d AD), racial interaction HAQ, racial interaction 3d AD, average energy density, average caloric intake, average dietary saturated fat percent, and average fiber intake; body composition: BMI and body fat percent. Lines represent stratified data from black (CCb) and white (CCw) girls and combined (CCc) data.

While the relationships between variables within the groups varied from year to year, the canonical correlation between the groups (obesity correlates and body composition) decreased with each subsequent visit as shown in Figure 3; the decreases in correlation followed linear trends with *R* ^2^ = 0.72 for the combined data, *R* ^2^ = 0.93 for black girls, and *R* ^2^ = 0.75 for white girls. Thisindicates an age-related reduction in association between obesity correlates and body composition in spite of an age-related increase in correlation for some of the variables in the bivariate results.

## 4. Discussion

In this study, physical activity was positively associated with diet quality indicators as early as 12 years of age (visit year 3) (Figures 1 and 2, Table 1). Furthermore, the increasing strength of correlation between diet quality indicators and physical activity during the study period indicates an increasingly tight association between these health behaviors with age (Figures 1 and 2). In evaluating correlates of obesity, white race and higher household income, habitual activity, and fiber intake were associated with having a lower BMI and body fat percent. The consistent correlation of race and income with obesity in the CCA (Table 2) supports that socioeconomic factors are a major consideration in obesity development which has been previously reported [18]. The age-related decline in canonical correlation of BMI and body fat percent with both health behaviors and socioeconomic factors (Figure 3) demonstrates a decreased connection between obesity correlates and body composition during transition into adulthood.

### 4.1. Physical Activity and Diet Interactions

The decreasing association between body composition and correlates of obesity during adolescence is contrasted by the increasingly tight relationship between physical activity and select predictors of diet quality with age. Similar to our findings of associations between diet quality indicators and physical activity, Gillman et al. [16] found that low fruit and vegetable intake was correlated with sedentary behaviors and more physical activity was related to lower saturated fat, trans fat, and cholesterol intake in a diverse cohort of 1322 men and women. Pate et al. [15] also found more physical activity to be positively associated with healthy diet choices as well as with other health behaviors like choosing not to smoke in the 1990 Youth Risk Behavior Survey of  11631 high school students suggesting that health behaviors have strong associations as early as high school. Our findings indicate that the association between physical activity and diet emerges already at 12 years of age (visit 3) and that the correlation between these health behaviors increases as adolescents mature.

While preference shapes health behaviors, physiological mechanisms like fatty acid oxidation may also influence the association between diet and activity because it responds to both dietary intake and physical activity [23]. High fat intake is associated with low carbohydrate intake and vice versa, and fatty acid oxidation increases with higher fat and lower carbohydrate intake [24–26]; exercise also increases fatty acid oxidation in muscle tissue performing or adapting to physical activity [23, 27]. Since high fat/low carbohydrate intake and physical activity have a similar physiological impact, accelerated fatty acid oxidation in response to physical activity may be reflexively counteracted by selection of a diet with low fat and energy density; low dietary fat intake suppresses fatty acid oxidation because it is generally higher in carbohydrate proportion [23, 26]. Conversely, decreasing physical activity decreases fatty acid oxidation [28]. As higher fat/lower carbohydrate proportion in dietary intake accelerates fatty acid oxidation [26], oxidation decrease by decreasing physical activity level could be innately balanced by intake of a diet higher in fat and energy density. We found an increasing negative correlation between physical activity and dietary fat measures with age while physical activity declines (Figure 1(a)). This increase in negative correlation falls in line with potential physiological associations between these health behaviors as well as with the existing support for health behavior patterns.

### 4.2. Obesity during Growth

Along with increasing strength of association between health behaviors with age, the decrease in canonical correlation between obesity correlates and body composition indicates a potential for higher impact of behaviors on anthropometrics at younger ages. The age-related disconnect between obesity correlates and body composition that we saw is supported by a study demonstrating that various fat deposits are genetically distinct “miniorgans” subject to developmental processes and maturation [29]. Similarly, a four-year study of adipose tissue growth in 288 children found that fat tissue development in children played a role in developing enlarged fat depots in obese adults [30]. The decrease in canonical correlation between obesity correlates and body composition with age in our study may well reflect fat storage capacity maturing during adolescence.

In addition to, or as a consequence of, developmental effects on fat tissue, juvenile obesity increases adult obesity risk. A 35-year longitudinal British study ending in 2000 demonstrated that early weight gain predicted adult obesity [31], and a 1976 study found 2.4 odds' ratio for adult obesity associated with juvenile obesity and overweight [32]. The alarming increase in obesity in the juvenile population today consequently bodes ill for obesity and subsequent health odds for the generation to come [1, 8, 13, 33]. These bleak odds, in conjunction with our findings, stress the importance of attention to health habits in children when the framework of health behaviors is less settled and body composition is more responsive.

### 4.3. Limitations

Limitations of this analysis include self-report of physical activity which tends to overestimate actual activity levels particularly at lower levels of activity [5]. The fluctuation in correlations at years seven and eight (Figures 1, 2, and 3) may be an artifact of the change in questionnaire collection protocol at year seven [20]. Another limitation of this study is the inclusion of all beverages in the energy density calculation necessitated by the structure of the data set. Energy density depends on the water content in the food as water adds weight but no calories [11, 34, 35], and separate calculations for food only, and food and select beverages are typically used to adjust for energy density differences in liquid and solid intake [34, 36, 37]. The effect of these limitations is an underestimation of dietary energy density due to the low energy density in liquids and a reduced range for differences in activity. Future studies using data from an electronic physical activity monitor and dietary assessment coded for removal of beverages for multiple energy density calculations would better identify the extent of the trends uncovered here. Longitudinal data from a broader age group could additionally determine if our discovered trends continue with age guiding obesity prevention in the general population.

### 4.4. Conclusion

Not only did our study confirm positive associations between physical activity and diet quality indicators in adolescent girls, but we also documented an increasingly tight correlation between these two health behaviors with age. As a consequence, successful interventions to increase activity all the way through adolescence may have positive effects on diet quality as behavior patterns are established. While physical activity and diet become more tightly correlated with age, body composition becomes less associated with known obesity correlates. The age-related decrease in association between factors that affect obesity and measures of obesity is consistent with findings that predictions of adult overweight using adolescent weight status improve with age [3]. The increasing predictability of future weight with age and our finding of increasing disconnect between obesity correlates and body composition during adolescence indicate that maturation of fat tissues during growth may impact weight status throughout adult life. Fat tissue maturation, high adult obesity odds for overweight youth, and the increasing correlation between health behaviors with age should spur investments in interventions to promote healthy diet and physical activity behaviors continuously through adolescence when the potential for a return on the investment through long term impact on obesity odds and future health is greatest.

## Supplementary Material

The supplementary files contain tables that are not in the manuscript for publication to help readers verify findings. The files consist of complete bivariate results as well as tables of canonical results stratified by race. It also contains the full version of the reduced tables of combined canonical result that are included in the manuscript. The tables were reduces for the manuscript by pulling out non-significant confounders to avoid the tables becoming too large.

## Figures and Tables

**Table 1:** Canonical correlation analysis evaluating physical activity and confounders with diet quality indicators. Percent variance explained by first indices and first indices' standardized canonical coefficients by visit year.

y	*n*	% var.	Physical activity, confounders						Diet quality			
			Inc	Race	RxHAQ	Rx3d AD	HAQ	3d AD	ED	Av kcal	Sat fat %	Fiber
3	1701	66.26	0.32^*∗∗*^	−0.64^*∗∗*^	0.14^*∗*^	−0.66^*∗∗*^	−0.03^†^	0.45^*∗*^	−0.88	−0.55	0.35	0.23
5	1576	66.19	0.41^*∗∗*^	−0.67^*∗∗*^	0.35^*∗*^	−0.54^*∗∗*^	−0.09^*∗∗*^	0.27^*∗∗*^	−0.67	−0.79	0.10	0.76
7	1457	69.97	0.27^*∗∗*^	−0.59^*∗∗*^	−0.10^*∗∗*^	−0.46^*∗∗*^	0.37^*∗∗*^	0.30^*∗∗*^	−0.57	−0.89	−0.15	0.91
8	1423	70.60	0.36^*∗∗*^	−0.41^*∗∗*^	−0.56^*∗∗*^	−0.31^*∗∗*^	0.89^*∗∗*^	0.29^*∗∗*^	−0.54	−0.65	−0.08	0.86
10	1699	74.26	0.36^*∗∗*^	−0.48^*∗∗*^	−0.33^*∗∗*^	−0.17^*∗∗*^	0.72^*∗∗*^	0.25^*∗∗*^	−0.60	−0.49	−0.17	0.65

^†^Significance at *P* < 0.1; ^*∗*^significance at *P* < 0.05; ^*∗∗*^significance at *P* < 0.001; for dependent variates significance is evaluated with a univariate *F* test for association with first indices canonical correlation.

3-day activity diary score, 3d AD; average caloric intake, Av kcal; average dietary saturated fat percent, Sat fat %; average energy density, ED; average fiber intake, fiber; habitual physical activity questionnaire score, HAQ; income category, Inc; percent variance, % var.; race by 3-day AD, Rx3d AD; race by HAQ, RxHAQ; year, y.

**Table 2:** Canonical correlation analysis evaluating health behaviors and confounders (obesity correlates) with body composition. Percent variance explained by first indices and first indices' standardized canonical coefficients by visit year.

y	*n*	% var.	Obesity correlates										Body composition	
			Inc	Race	RxHAQ	Rx3d AD	HAQ	3d AD	ED	Av kcal	Sat fat %	Fiber	BMI	BF%
3	1693	81.43	0.21^*∗∗*^	−0.50^*∗∗*^	0.33^*∗∗*^	0.11^*∗∗*^	−0.36^*∗∗*^	−0.29^*∗∗*^	0.07^*∗*^	0.05^*∗*^	−0.21^*∗∗*^	−0.05^*∗∗*^	−1.80	1.12
5	1557	75.28	0.38^*∗∗*^	−0.32^*∗∗*^	0.01^*∗∗*^	−0.45^*∗∗*^	0.06^*∗∗*^	0.18^*∗∗*^	0.16^*∗*^	0.30^*∗∗*^	−0.12^*∗*^	−0.26^*∗*^	−1.61	0.75
7	1412	62.65	0.32^*∗∗*^	−0.71^*∗∗*^	0.24^*∗∗*^	0.14^*∗*^	−0.04^*∗∗*^	−0.22	0.18	−0.02^*∗*^	0.28^†^	0.26^*∗∗*^	−1.19	0.22
8	1378	63.45	0.15^*∗∗*^	−0.67^*∗∗*^	0.37^*∗∗*^	−0.64^*∗∗*^	−0.19^*∗∗*^	0.18^*∗∗*^	0.18	0.07^*∗*^	0.17^*∗*^	0.03^*∗*^	−1.73	0.90
10	1679	83.47	0.38^*∗∗*^	−0.68^*∗∗*^	−0.11^*∗∗*^	0.11^*∗∗*^	0.42^*∗∗*^	−0.28^*∗*^	0.32	0.14	0.08	0.06^*∗∗*^	−1.14	0.16

^†^Significance at *P* < 0.1; ^*∗*^significance at *P* < 0.05; ^*∗∗*^significance at *P* < 0.001; for dependent variates significance is evaluated with a univariate *F* test for association with first indices canonical correlation.

3-day activity diary score, 3d AD; average caloric intake, Av kcal; average dietary saturated fat percent, Sat fat %; average energy density, ED; average fiber intake, fiber; habitual physical activity questionnaire score, HAQ; income category, Inc; body fat percent, BF%; percent variance, % var.; race by 3-day AD, Rx3d AD; race by HAQ, RxHAQ; year, y.

## References

[1] Ogden C. L., Carroll M. D., Kit B. K., Flegal K. M. (2012). Prevalence of obesity and trends in body mass index among US children and adolescents, 1999–2010. *The Journal of the American Medical Association*.

[2] Flegal K. M., Carroll M. D., Ogden C. L., Curtin L. R. (2010). Prevalence and trends in obesity among US adults, 1999–2008. *The Journal of the American Medical Association*.

[3] Guo S. S., Chumlea W. C. (1999). Tracking of body mass index in children in relation to overweight in adulthood. *American Journal of Clinical Nutrition*.

[4] US Department of Agriculture (2011). *Dietary Guidelines for Americans 2010*.

[5] Troiano R. P., Berrigan D., Dodd K. W., Mâsse L. C., Tilert T., Mcdowell M. (2008). Physical activity in the United States measured by accelerometer. *Medicine and Science in Sports and Exercise*.

[6] Andersen R. E., Crespo C. J., Bartlett S. J., Cheskin L. J., Pratt M. (1998). Relationship of physical activity and television watching with body weight and level of fatness among children: results from the Third National Health and Nutrition Examination Survey. *The Journal of the American Medical Association*.

[7] Simon C., Schweitzer B., Oujaa M. (2008). Successful overweight prevention in adolescents by increasing physical activity: a 4-year randomized controlled intervention. *International Journal of Obesity*.

[8] Andersen L. B., Harro M., Sardinha L. B. (2006). Physical activity and clustered cardiovascular risk in children: a cross-sectional study (The European Youth Heart Study). *The Lancet*.

[9] Berkey C. S., Rockett H. R. H., Gillman M. W., Colditz G. A. (2003). One-year changes in activity and in inactivity among 10- to 15-year-old boys and girls: relationship to change in body mass index. *Pediatrics*.

[10] Lichtenstein A. H., Appel L. J., Brands M. (2006). Diet and lifestyle recommendations revision 2006: a scientific statement from the American Heart Association Nutrition Committee. *Circulation*.

[11] Ledikwe J. H., Blanck H. M., Khan L. K. (2006). Low-energy-density diets are associated with high diet quality in adults in the United States. *Journal of the American Dietetic Association*.

[12] Kant A. K., Graubard B. I. (2005). Energy density of diets reported by American adults: association with food group intake, nutrient intake, and body weight. *International Journal of Obesity*.

[13] Troiano R. P., Briefel R. R., Carroll M. D., Bialostosky K. (2000). Energy and fat intakes of children arid adolescents in the United States: Data from the National Health and Nutrition Examination Surveys. *American Journal of Clinical Nutrition*.

[14] Krebs-Smith S. M., Guenther P. M., Subar A. F., Kirkpatrick S. I., Dodd K. W. (2010). Americans do not meet federal dietary recommendations. *Journal of Nutrition*.

[15] Pate R. R., Heath G. W., Dowda M., Trost S. G. (1996). Associations between physical activity and other health behaviors in a representative sample of US adolescents. *American Journal of Public Health*.

[16] Gillman M. W., Pinto B. M., Tennstedt S., Glanz K., Marcus B., Friedman R. H. (2001). Relationships of physical activity with dietary behaviors among adults. *Preventive Medicine*.

[17] Lytle L. A., Seifert S., Greenstein J., McGovern P. (2000). How do children's eating patterns and food choices change over time? Results from a cohort study. *American Journal of Health Promotion*.

[18] NHLBI Research Group (1992). Obesity and cardiovascular disease risk factors in black and white girls: the NHLBI Growth and Health Study. *American Journal of Public Health*.

[19] Kimm S. Y. S., Glynn N. W., Kriska A. M. (2002). Decline in physical activity in black girls and white girls during adolescence. *The New England Journal of Medicine*.

[20] Kimm S. Y. S., Glynn N. W., Kriska A. M. (2000). Longitudinal changes in physical activity in a biracial cohort during adolescence. *Medicine and Science in Sports and Exercise*.

[22] IBM Corporation (2013). *IBM SPSS Statistics for Windows*.

[23] Smith S. R., De Jonge L., Zachwieja J. J. (2000). Concurrent physical activity increases fat oxidation during the shift to a high-fat diet. *American Journal of Clinical Nutrition*.

[24] Rolls B. J. (1995). Carbohydrates, fats, and satiety. *The American Journal of Clinical Nutrition*.

[25] Tremblay A., Plourde G., Despres J.-P., Bouchard C. (1989). Impact of dietary fat content and fat oxidation on energy intake in humans. *American Journal of Clinical Nutrition*.

[26] Prentice A. M. (1998). Manipulation of dietary fat and energy density and subsequent effects on substrate flux and food intake. *American Journal of Clinical Nutrition*.

[27] Starritt E. C., Angus D., Hargreaves M. (1999). Effect of short-term training on mitochondrial ATP production rate in human skeletal muscle. *Journal of Applied Physiology*.

[28] Holloszy J. O., Coyle E. F. (1984). Adaptations of skeletal muscle to endurance exercise and their metabolic consequences. *Journal of Applied Physiology Respiratory Environmental and Exercise Physiology*.

[29] Tchkonia T., Lenburg M., Thomou T. (2007). Identification of depot-specific human fat cell progenitors through distinct expression profiles and developmental gene patterns. *American Journal of Physiology—Endocrinology and Metabolism*.

[30] Knittle J. L., Timmers K., Ginsberg-Fellner F., Brown R. E., Katz D. P. (1979). The growth of adipose tissue in children and adolescents. Cross-sectional and longitudinal studies of adipose cell number and size. *Journal of Clinical Investigation*.

[31] Toschke A. M., Rückinger S., Reinehr T., von Kries R. (2008). Growth around puberty as predictor of adult obesity. *European Journal of Clinical Nutrition*.

[32] Rimm I. J., Rimm A. A. (1976). Association between juvenile onset obesity and severe adult obesity in 73, 532 women. *American Journal of Public Health*.

[33] Anderssen N., Jacobs D. R., Sidney S. (1996). Change and secular trends in physical activity patterns in young adults: a seven-year longitudinal follow-up in the Coronary Artery Risk Development in Young Adults study (CARDIA). *American Journal of Epidemiology*.

[34] Ledikwe J. H., Blanck H. M., Khan L. K. (2005). Dietary energy density determined by eight calculation methods in a nationally representative United States population. *Journal of Nutrition*.

[35] Kral T. V. E., Stunkard A. J., Berkowitz R. I., Stallings V. A., Brown D. D., Faith M. S. (2007). Daily food intake in relation to dietary energy density in the free-living environment: a prospective analysis of children born at different risk of obesity. *American Journal of Clinical Nutrition*.

[36] Kral T. V. E., Berkowitz R. I., Stunkard A. J., Stallings V. A., Brown D. D., Faith M. S. (2007). Dietary energy density increases during early childhood irrespective of familial predisposition to obesity: results from a prospective cohort study. *International Journal of Obesity*.

[37] Ello-Martin J. A., Roe L. S., Ledikwe J. H., Beach A. M., Rolls B. J. (2007). Dietary energy density in the treatment of obesity: a year-long trial comparing 2 weight-loss diets. *American Journal of Clinical Nutrition*.

