# Supplementary material for: Associations among Physical Activity, Diet, and Obesity Measures Change during Adolescence
Source: J Nutr Metab. 2015 Oct 11;2015:805065. doi: 10.1155/2015/805065 (PMC4619959; doi:10.1155/2015/805065)
Supplement: Supplementary file 1 — The supplementary files contain tables that are not in the manuscript for publication to help readers verify findings. The files consist of complete bivariate results as well as tables of canonical results stratified by race. It also contains the full version of the reduced tables of combined canonical result that are included in the manuscript. The tables were reduces for the manuscript by pulling out non-significant confounders to avoid the tables becoming too large. [file 805065.f1.pdf]

Supplemental Tables for Reviewers Only

TABLE S1. Bivariate results; Spearman's rho correlation for income, race, habitual physical activity questionnaire score (HAQ), 3 day activity diary score (3d AD), average energy density (ED), average caloric intake (Av kcal), average dietary saturated fat percent (Sat fat %), average fiber intake (fiber) by visit year

	Year	Income	Race	HAQ	3d AD	ED	Av kcal	Satfat%	Fiber	BMI	BF%
Income	3	1	-.348**	.069**	-.056	-.176**	-.007	-.090**	.057	-.114**	-.034
	5	1	-.358**	.122**	.036	-.164**	-.010	-.175**	.128**	-.147**	-.079**
	7	1	-.354**	.301**	.012	-.185**	-.053	-.156**	.132**	-.144**	-.135**
	8	1	-.348**	.289**	-.028	-.154**	-.074**	-.211**	.138**	-.149**	-.114**
	10	1	-.357**	.317**	-.067**	-.207**	-.083**	-.238**	.144**	-.172**	-.163**
Race	3		1	-.090**	-.088**	.208**	.088**	.020	-.022	.188**	.087**
	5		1	-.193**	-.104**	.250**	.099**	.079**	-.064**	.185**	.094**
	7		1	-.346**	-.164**	.300**	.133**	.120**	-.121**	.200**	.121**
	8		1	-.291**	-.075**	.276**	.103**	.142**	-.115**	.207**	.108**
	10		1	-.348**	-.013	.373**	.111**	.206**	-.119**	.207**	.162**
HAQ	3			1	.263**	-.038	.017	-.013	.042	-.060**	-.086**
	5			1	.285**	-.095**	.020	-.045	.075**	-.090**	-.120**
	7			1	.249**	-.153**	.007	-.138**	.199**	-.141**	-.152**
	8			1	.220**	-.129**	-.014	-.192**	.156**	-.128**	-.155**
	10			1	.103**	-.172**	-.066**	-.212**	.202**	-.144**	-.159**
AD	3				1	.004	.056	.004	.064**	.003	-.085**
	5				1	-.001	.082**	.062**	.075**	-.028	-.081**
	7				1	-.049	.086**	-.017	.127**	.003	-.005
	8				1	-.011	.096**	-.020	.136**	.052	.031
	10				1	-.071**	.050	-.047	.048	.078**	.062**
ED	3					1	.249**	.342**	.064**	-.029	-.077**
	5					1	.291**	.382**	.094**	-.056	-.084**
	7					1	.290**	.449**	.060	-.035	-.031
	8					1	.261**	.481**	.074**	-.053	-.045
	10					1	.238**	.458**	.037	-.024	.001
Kcal	3						1	.102**	.678**	-.046	-.097**
	5						1	.195**	.664**	-.127**	-.148**
	7						1	.209**	.627**	-.069**	-.089**
	8						1	.231**	.620**	-.066**	-.082**
	10						1	.221**	.575**	-.028	-.049
Satfat%	3							1	-.154**	.046	-.010
	5							1	-.068**	.025	.017
	7							1	-.114**	-.040	-.034

	8							1	-.093**	-.019	.000
	10							1	-.151**	.008	.016
Fiber	3								1	-.092**	-.123**
	5								1	-.113**	-.118**
	7								1	-.102**	-.117**
	8								1	-.089**	-.076**
	10								1	-.088**	-.095**
BMI	3									1	.835**
	5									1	.853**
	7									1	.864**
	8									1	.873**
	10									1	.900**

** significance at $P < 0.001$; two-tailed t test

TABLE S2. Percent variance (% var.) explained by first indices and first indices' canonical correlation (CC). Standardized canonical coefficients for physical activity and confounders [income category (Inc), race, maturation stage (MS), height (Ht), weight (Wt), race by HAQ (RxHAQ), race by 3 day AD (Rx3d AD), habitual physical activity questionnaire score (HAQ), 3 day activity diary score (3d AD)],and diet quality indicators [average energy density (ED), average caloric intake (Av kcal), average dietary saturated fat percent (Sat fat %), average fiber intake (fiber)] by visit year (y).

				Physical Activity, Confounders									Diet Quality			
y	n	% var.	CC	Inc	Race	MS	Ht	Wt	Rx HAQ	Rx3d AD	HAQ	3d AD	ED	Av kcal.	Sat fat %	fiber
3	1701	66.26	0.29	0.32**	-0.64**	0.32	-0.30*	0.29†	0.14*	-0.66**	-0.03†	0.45*	-0.88	-0.55	0.35	0.23
5	1576	66.19	0.34	0.41**	-0.67**	0.02*	-0.18*	0.38	0.35*	-0.54**	-0.09**	0.27**	-0.67	-0.79	0.10	0.76
7	1457	69.97	0.42	0.27**	-0.59**	-0.02*	-0.06*	0.10*	-.10**	-0.46**	0.37**	0.30**	-0.57	-0.89	-0.15	0.91
8	1423	70.60	0.41	0.36**	-0.41**	-0.00	-0.04**	0.16	-0.56**	-0.31**	0.89**	0.29**	-0.54	-0.65	-0.08	0.86
10	1699	74.26	0.46	0.36**	-0.48**	n/a	-0.08*	0.16*	-0.33**	-0.17**	0.72**	0.25**	-0.60	-0.49	-0.17	0.65

† significance at P < 0.1; * significance at P < 0.05; ** significance at P < 0.001; for dependant variates significance is evaluated with a univariate F test for association with first indices canonical correlation.

TABLE S3. Cohort stratified by race (black). Percent variance (% var) explained by first indices and first indices' canonical correlation (CC). Standardized canonical coefficients for physical activity and confounders [income category (Inc), maturation stage (MS), height (Ht), weight (Wt), habitual physical activity questionnaire score (HAQ), 3 day activity diary score (3d AD)],and diet quality indicators [average energy density (ED), caloric intake (Av kcal), average dietary saturated fat percent (Sat fat %), average fiber intake (fiber)] by visit year (y).

				Physical Activity, Confounders						Diet Quality			
y	n	% var.	CC	Inc	MS	Ht	Wt	HAQ	3d AD	ED	Av kcal	Sat fat%	fiber
3	870	67.24	0.22	0.23*	0.48	-0.50	0.40	0.00	-0.70**	-0.43	-0.39	0.11	-0.53
5	815	70.17	0.29	0.72**	-0.02	-0.21	0.49*	0.37*	-0.47**	-0.37	-0.91	-0.26	0.80
7	741	52.78	0.24	0.38*	0.01†	-0.25*	0.44*	0.21*	-0.79**	-0.31	-0.81	-0.21	0.02
8	734	57.52	0.23	0.51**	0.09	-0.35†	0.65†	-0.00	-0.62**	-0.46	-0.70	-0.19	-0.00
10	861	56.70	0.25	0.72**	n/a	-0.11	0.49†	0.19*	-0.41**	-0.12	-0.72	-0.58	0.19

† significance at P < 0.1; * significance at P < 0.05; ** significance at P < 0.001; for dependant variates significance is evaluated with a univariate F test for association with first indices canonical correlation.

TABLE S4. Cohort stratified by race (white). Percent variance (% var) explained by first indices and first indices' canonical correlation (CC). Standardized canonical coefficients for physical activity and confounders [income category (Inc), maturation stage (MS), height (Ht), weight (Wt), habitual physical activity questionnaire score (HAQ), 3 day activity diary score (3d AD)], and diet quality indicators [average energy density (ED), caloric intake (Av kcal), average dietary saturated fat percent (Sat fat %), average fiber intake (fiber)] by visit year (y).

				Physical Activity, Confounders						Diet Quality			
y	n	% var.	CC	Inc	MS	Ht	Wt	HAQ	3d AD	ED	Av kcal	Sat fat%	fiber
3	831	48.80	0.17	0.86**	0.17*	0.35*	-0.10*	0.14	-0.10	-0.79	0.33	-0.30	0.15
5	761	62.86	0.27	0.71**	-0.39*	0.37†	-0.35*	-0.01†	-0.00*	0.22	0.41	-0.69	0.41
7	716	77.85	0.34	0.59**	-0.04	0.02†	-0.01†	0.69**	0.02†	0.08	-0.15	-0.38	0.93
8	689	71.13	0.36	0.53**	0.07	0.16**	-0.09	0.65**	0.12*	0.05	-0.26	-0.61	0.78
10	838	80.26	0.38	0.55**	n/a	-0.01**	0.05	0.73**	0.06*	-0.11	-0.32	-0.45	0.79

† significance at P < 0.1; * significance at P < 0.05; ** significance at P < 0.001; for dependant variates significance is evaluated with a univariate F test for association with first indices canonical correlation.

TABLE S5. Percent variance (% var.) explained by first indices and first indices' canonical correlation (CC). Standardized canonical coefficients for obesity correlates [income category (Inc), race, maturation stage (MS), height (Ht), weight (Wt), race by HAQ (RxHAQ), race by 3 day AD (Rx3d AD), habitual physical activity questionnaire score (HAQ), 3 day activity diary score (3d AD), average energy density (ED), average caloric intake (Av kcal), average dietary saturated fat percent (Sat fat %), average fiber intake (fiber)], and body composition [BMI, body fat percent (BF%)] by visit year (y).

				Obesity Correlates											Body Composition	
y	n	% var.	CC	Inc	Race	MS	Rx HAQ	Rx3d AD	HAQ	3d AD	ED	Av kcal	Sat fat %	fiber	BMI	BF%
3	1693	81.43	0.39	0.21**	-0.50**	-0.77**	0.33**	0.11**	-0.36**	-0.29**	0.07*	0.05*	-0.21**	-0.05**	-1.80	1.12
5	1557	75.28	0.35	0.38**	-0.32**	-0.56**	0.01**	-0.45**	0.06**	0.18**	0.16*	0.30**	-0.12*	-0.26*	-1.61	0.75
7	1412	62.65	0.27	0.32**	-0.71**	-0.27**	0.24**	0.14*	-0.04**	-0.22	0.18	-0.02*	0.28†	0.26**	-1.19	0.22
8	1378	63.45	0.30	0.15**	-0.67**	-0.11*	0.37**	-0.64**	-0.19**	0.18**	0.18	0.07*	0.17*	0.03*	-1.73	0.90
10	1679	83.47	0.29	0.38**	-0.68**	n/a	-0.11**	0.11**	0.42**	-0.28*	0.32	0.14	0.08	0.06**	-1.14	0.16

† significance at P < 0.1; * significance at P < 0.05; ** significance at P < 0.001; for dependant variates significance is evaluated with a univariate F test for association with first indices canonical correlation.

TABLE S6. Cohort stratified by race (black). Percent variance (% var) explained by first indices and first indices' canonical correlation (CC). Standardized canonical coefficients for obesity correlates [income category (Inc) maturation stage (MS), height (Ht), weight (Wt), habitual physical activity questionnaire score (HAQ), 3 day activity diary score (3d AD), average energy density (ED), average caloric intake (Av kcal), average dietary saturated fat percent (Sat fat %), average fiber intake (fiber)], and body composition [BMI, body fat percent (BF%)] by visit year (y).

				Obesity Correlates								Body Composition	
y	n	% var.	CC	Inc	MS	HAQ	3d AD	ED	Av kcal	Sat fat %	fiber	BMI	BF%
3	865	63.89	0.29	0.25**	-0.92**	0.02	-0.24*	0.07*	0.09*	-0.31*	-0.08†	-1.91	1.27
5	802	55.82	0.26	0.02**	0.66**	-0.43*	0.02**	-0.31*	-0.57*	-0.05*	0.37	-0.50	1.43
7	714	89.14	0.25	0.44*	-0.45*	0.50*	-0.10	0.09	0.17*	0.29†	0.32*	0.81	-1.66
8	701	56.55	0.23	0.32*	0.05	0.70**	-0.26*	0.30†	0.66*	-0.34*	-0.18†	0.72	-1.60
10	851	75.32	0.18	0.43*	n/a	0.36*	-0.31	0.53**	0.35†	0.19†	0.12	-0.70	-0.32

† significance at P < 0.1; * significance at P < 0.05; ** significance at P < 0.001; for dependant variates significance is evaluated with a univariate F test for association with first indices canonical correlation.

TABLE S7. Cohort stratified by race (white). Percent variance (% var) explained by first indices and first indices' canonical correlation (CC). Standardized canonical coefficients for obesity correlates [income category (Inc) maturation stage (MS), height (Ht), weight (Wt), habitual physical activity questionnaire score (HAQ), 3 day activity diary score (3d AD), average energy density (ED), average caloric intake (Av kcal), average dietary saturated fat percent (Sat fat %), average fiber intake (fiber)], and body composition [BMI, body fat percent (BF%)] by visit year (y).

				Obesity Correlates								Body Composition	
y	n	% var.	CC	Inc	MS	HAQ	3d AD	ED	Av kcal	Sat fat %	fiber	BMI	BF%
3	828	81.76	0.39	0.22*	-0.84**	-0.20**	-0.23**	0.09	0.05	-0.23*	-0.09*	-1.76	1.17
5	755	88.34	0.36	0.51**	-0.70**	0.12*	-0.02*	0.28*	0.13*	-0.21	-0.06*	-1.14	0.17
7	698	66.57	0.24	0.57**	-0.43*	0.40**	-0.13	0.30*	0.01	0.04*	0.22*	-0.26	-0.76
8	677	76.35	0.27	0.47**	-0.40*	0.56**	-0.29	0.30	0.17	-0.15	0.10	0.11	-1.10
10	828	80.54	0.26	0.70**	n/a	0.56**	-0.28	0.10*	0.12	0.03	0.02*	0.51	0.52

† significance at P < 0.1; * significance at P < 0.05; ** significance at P < 0.001; for dependant variates significance is evaluated with a univariate F test for association with first indices canonical correlation
